# Formulating a return-to-work decision for employees with major depressive disorders: occupational therapists’ experiences

**DOI:** 10.4102/phcfm.v8i2.954

**Published:** 2016-04-20

**Authors:** Enos Ramano, Tania Buys, Marianne de Beer

**Affiliations:** 1 Occupational Therapist, South Africa; 2 Department of Occupational Therapy, School of Health Care Sciences, Faculty of Health Sciences, University of Pretoria, South Africa

## Abstract

**Background:**

Major depressive disorder (MDD) is worldwide one of the most concerning health problems as it is associated with reduced work productivity and permanent disability. Occupational therapists are often called upon to make a return-to-work decision on employees with MDD in order to facilitate continued employment. Sustaining employment is in alignment with achieving the Millennium Development Goal 1: Eradicating extreme poverty, as it is known that people suffering from mental health disorders are frequently denied employment opportunities leading to reduced financial resources and therefore possible poverty.

**Aim:**

This study described occupational therapists’ experiences of formulating a return-to-work decision on employees with MDD. It formed part of a larger study.

**Setting:**

Occupational therapists working in vocational rehabilitation or mental health in South Africa with a postgraduate qualification in vocational rehabilitation or mental health participated in the study.

**Method:**

A qualitative research design was used. Two separate focus groups explored 11 occupational therapists’ experiences of formulating a return-to-work decision on employees with MDD. Ethics clearance number: S34/2007.

**Results:**

Seven themes emerged, which were, (1) the biographical profile of the employee, (2) point of view of employer, (3) point of view of employee, (4) point of view of occupational therapist, (5) declaring the employee as temporary incapacitated, (6) declaring the employee as permanently incapacitated and (7) employee’s level of motivation.

**Conclusion:**

Occupational therapists ought to have sound knowledge, skill, experience and the ability to collaborate with employees and employers in formulating a return-to-work decision.

## Introduction

The World Health Organisation (WHO) estimates major depressive disorder (MDD) to be the leading burden of disease by 2030.^1^ The lifetime prevalence of depression is in the range of 10 to 15% in the general population^1^ and is associated with substantial disability.^2^ Studies by Gold and Shuman^3^ showed that MDD is one of the leading causes of disability amongst adults in the world. Without intervention, depression has a tendency to be chronic, recurrent and to be associated with increasing disability and decline in the quality of life.^2^

Skeen, Lund, Kleintjes, Flisher^4^ as well as Miranda and Patel^5^ indicate in work conducted with reference to the Millennium Development Goals (MDGs) that there are associations with poverty and poor mental health, thus sound work evaluations, decision-making and intervention in the return-to-work process are critical in maintaining suitable employment for employees with MDDs. This will contribute towards preventing the possibility of employees gradually becoming financially more restricted with resultant increased poverty levels, despite possible access to insured group disability benefits. This could occur as medical costs related to the MMD are high and access to public health care facilities for the treatment of mental health conditions is limited. Loss of income from a salary, combined with costs related to the treatment of the condition, role changes within the family structure as well as limited implementation of disability equity law could result in increased poverty amongst people living with various mental health conditions, including that of MDD.

Major depression presents in diverse ways as it varies across age, gender, culture and medical settings. According to the Diagnostic Statistical Manual-5 (DSM-5), for a diagnosis of MDD, symptoms must last at least two weeks and, typically, a person will experience at least four symptoms. These include changes in appetite and weight, changes in sleep and activity, lack of energy, feelings of guilt, problems thinking and making decisions, and recurring thoughts of death or suicide.^6^ Major depression results in work absenteeism and, even more importantly, loss of productivity whilst the employee is present at work.^2^ Often unrecognised and untreated, MDD have far reaching economic consequences.

Occupational therapists play a key role in facilitating safe, effective and timely return-to-work, as this is hailed as an important part of an individual’s recovery.^7^ One way of determining whether an employee is able to-return-to-work after suffering an illness such as MDD is to conduct a functional capacity evaluation,^8^ the outcome of which could also be used for compensation entitlement^8^ to support claims or benefits application,^9^ to explore alternative career options^8^ and as a basis for recommending modifications to the individual’s workstation or workplace.^9^

The challenge that faces occupational therapists, however, is that there is no specific guideline on a return-to-work decision.

Because functional disability is a risk factor for MDD, the possibility of preventing or treating MDD in rehabilitation settings should be explored. This indicates the need for clinical evidence, research and clarifications from occupational therapists regarding their assessments, intervention and rehabilitation programmes involving employees suffering from MDD.

The aim of this part of the study was to describe the occupational therapists’ experiences of formulating a return-to-work decision on employees with MDD’s.

## Method and design

### Study design

A qualitative design with an interpretive paradigm was used to determine the participants’ inter-subjective experiences of formulating a return-to-work decision. The researcher observed the context in which participants’ experiences occurred and gave them the opportunity to reflect on these experiences in their own words.^10^

### Setting

The study population included occupational therapists registered with the Health Professions Council of South Africa and working in the field of vocational rehabilitation or mental health and who had postgraduate qualifications in occupational therapy (vocational rehabilitation or mental health).

### Study population and sampling strategy

Purposive sampling was used to strategically judge and select participants most experienced in the phenomenon studied and who were able to articulate and explain their experiences to the researcher.^11,12^

### Data collection

Two focus group interviews were conducted with 11 occupational therapists at the university campus where the researcher is a part-time lecturer. Focus groups allowed the researcher to distinguish similarities and differences in the participants’ views through group interaction.^13^ The focus groups lasted between 90 to 120 minutes and were recorded electronically. See Box 1 for the interview guide.

**BOX 1:** Focus group interview guide.

**Work capacity:** If you think about the role of the occupational therapist in determining work capacity what comes to mind?
**Work incapacity:** Which factors will you take into consideration to declare clients as unable to work?
**Malingering:** Do you sometimes find that clients suffering from a major depressive disorder pretend that they are ill in order to avoid returning to work?
**Personality disorders:** To what extent do you think an additional diagnosis will influence a client’s capacity and ability to-return-to-work?
**Practices of occupational therapists:** What do you perceive or view as a good occupational therapy practice in determining work capacity with a client suffering from a major depressive disorder?
Is it possible to give some specific criteria for a good occupational therapy practice?
**Steps to determine work capacity:** Occupational therapists use different steps to determine work capacity with clients suffering from major depressive disorder. How will you describe the steps that you think a competent occupational therapist should follow to perform a quality type of functional capacity evaluation?

*Source*: Ramano EM^14^

Three senior lecturers, who are experts in qualitative studies, scrutinised the questions before they were used for the focus group interviews.

Open-ended questions were used to encourage reflection and rich description of ideas and experiences. Participants were encouraged to elaborate on their answers using their experiences and examples.^15^

### Data analysis

The data from the two focus groups, which were verbatim transcribed, were analysed, coded and categorised. Through this process a thematic analysis was carried out and themes emerged as outlined by Paterson and Higgs 2005.^15^ The researcher and an external auditor analysed the data to enhance trustworthiness. On completion of both analyses, themes were discussed for consensus.

### Ethical considerations

Ethics approval was obtained from the Health Sciences Research Ethics Committee (S34/2007) at the University where the study was conducted.

Each participant received, and completed, a written consent form prior to their participation in one of the focus groups.

## Results

Seven themes emerged from the focus groups: (1) the biographical profile of the employee, (2) point of view of employer, (3) point of view of employee, (4) point of view of occupational therapist, (5) declaring the employee as temporary incapacitated, (6) declaring the employee as permanently incapacitated and (7) employee’s level of motivation. Each theme is detailed below with representative quotes from the data to illustrate the key features of the theme.

### Theme one: Biographical profile of the employee

All participants agreed that age, gender and type of job have an effect in formulating a return-to-work decision. All three of these factors contributed towards the decision, but participants could not quantify the effect of each as they were inter-related. Depending on the context, age, gender and type of work performed played a role in whether employees could return-to-work. This notion was supported by one of the participants who said: ‘… the [*employee’s*] age, gender and the type of job have an effect in returning them to work …’ (Participant 1, female, 43).

### Theme two: Employer’s view point

Understanding the perspective of the employer and aspects related to the employment situation were important considerations in formulating the decision to-return-to-work. These included:

- The employee’s previous work history – whether there was a stable or interrupted work history and whether there was a prior history of poor work performance.
- Employers’ ignorance about MDD.
- The employer’s unwillingness to reasonably accommodate the employee or implement work place adaptations.
- A ‘hostile’ workplace resulting in workplace barriers that are difficult to overcome.
- The possibility of constructive dismissal where employees resign as they feel there are no other options open to them. This decision is frequently made because of an intolerable workplace and is compounded by diagnosis-related limitations. One participant stated that:
  ‘The employer is the judge of reasonable accommodation and you [*therapist*] will need to negotiate with the employer …’ (Participant 2, female, 50).

### Theme three: Employee’s view point

A number of factors were found to have an impact on the employee’s perspective and their view on whether they could return-to-work. These factors would become evident during the functional capacity evaluation and could be attributed to, (1) the employee’s fear of returning to work, (2) stigma associated with their MDD condition or mental health, (3) lack of work skill affecting work performance, (4) poor work motivation and (5) secondary gain obtained whilst not at work, such as continuing to receive an income whilst not productively employed. One participant was of the opinion that, ‘It is difficult to return the client [*employee*] to work when they [*employee*] fear returning to work.’ (Participant 11, female, 36).

### Theme four: Occupational therapist’s view point

Two sub-themes emerged from the data which contributed towards the occupational therapist decision-making. These were grouped as ‘the medical decision’ and ‘the pyschosocial decision’. Inter-related participants were of the opinion that there were two distinct types of factors which influenced their return-to-work decision.

Seven aspects were identified as contributing to the medical decision, (1) degree of major depression, (2) impact of other factors on the diagnosis of MDD, such as comorbid conditions and environmental or contextual factors, (3) affected occupational performance areas, (4) rehabilitation potential (functional prognosis), (5) employee’s work endurance (stamina), (6) psychiatrist’s support to the employee and the return-to-work process and (7) employees time off work. One participant stated in this respect, ‘… be sure that the client can keep up with the job …’ (Participant 1, female, 43).

Eight key factors contributed to the psychosocial decision, (1) social expectations at work, (2) impact on interpersonal relationship at work, (3) support system at work, home or health team, (4) previous attempts at returning to work, (5) availability of work preparation or intervention programmes, (6) employee’s residual transferable skills or potential for re-skilling, (7) opportunity for the implementation of work trials (three months supported work trial) and previous participation in these trials and (8) assessment of the job and work environment. One participant commented as follows:

‘… match the client [*employee*] with the work … before you [*therapist*] can even return the client [*employee*] to work … you [*therapist*] need to know what to expect from work.’ (Participant 7, female, 54)

### Theme five: Declaring the employee as temporary incapacitated

This theme related to factors which would contribute to the occupational therapist making recommendations to the referring company or employer and which would assist them in making a decision of ‘*temporary incapacity*’ aligned with the provisions of relevant South African labour and insurance law. One of the outcomes of this process would be that the employee be granted extended time off work whilst receiving disability benefits, thereby facilitating intervention and return-to-work. Participants identified the following factors as impacting this decision, (1) stage of recovery, (2) opportunities for facilitation of return-to-work such as case management, (3) management of external or contextual factors affecting return-to-work, (4) reasonable accommodations or work place adaptation options, (5) optimal treatment obtained and (6) return-to-work program implementation possibilities. A number of participants alleged that:

‘We [*therapists*] have to remember to leave the door open to allow the client [*employee*] to-return-to-work at some stage and not put them [*employees*] at home permanently on a disability grant …’ (Participant 6, female, 46).

‘… match the client [*employee*] with the work … before you [*therapist*] can even return the client [*employee*] to work … you [*therapist*] need to know what to expect from work.’ (Participant 7, female, 54).

### Theme six: Declaring the employee as permanently incapacitated

Data collected during the process of functional capacity evaluation could culminate in the occupational therapist presenting evaluation results which would assist the referring company to consider a decision of permanent incapacity of the employee. A decision of permanent incapacity has a negative impact on the employee’s well-being as they will likely never return to their workplace or reasonable alternative productive open labour market employment. Employees who have been on extended sick or incapacity leave for a number of years lose their identities as workers, thereby reducing the likelihood of returning to work. Factors identified by the participants as contributing to this decision were, (1) poor medical prognosis, (2) poor functional prognosis, (3) poor coping skills despite support, (4) poor work productivity following intervention, (5) no reasonable accommodation or workplace adaptation opportunities. One participant stated that, ‘… long sick leave … it is difficult to return them [*employees*] back to work …’ (Participant 6, female, 46).

### Theme seven: Employee’s level of motivation

Participants were of the opinion that the employee’s level of motivation would impact on the employee’s ability to-return-to-work and, therefore, contribute towards the return-to-work decision. Factors identified by the participants were, (1) intrinsic motivation, (2) extrinsic motivators were seen as access to transport, family support and financial resources, (3) the employee’s level of motivation according to the Vona du Toit Model of Creative Ability (VdTMoCA), (4) work environmental factors such as workplace disciplinary enquiries, retrenchment policies and practices and work related stress, (5) the intensity of reluctance to-return-to-work (‘work phobia’) and (6) being off work for very extended periods of time. Participants stated:

‘… patients [*employees*] who have been off work for a long period of time … six months or more … their motivation to-return-to-work are mostly affected …’ (Participant 9, female, 50).

## Discussion

The results provide relevant insights into practicing occupational therapists’ experiences of formulating a return-to-work decision. Overall, the results indicate that practicing occupational therapists’ perceive this decision as a multifacetted and complex task influenced by a number of inter-related factors. Data emerging from this research process was generally supported by published research results.

The biographical profile of the employee theme is reported in literature as being a strong predictor of return-to-work with gender the strongest predictor.^4^ Socio-demographic variables such as gender and age are important factors for the ability to-return-to-work.^16,17^ Being male and young amongst those who were sick were predictors of RTW, as men returned to work earlier after a certified sick leave than women.^16^ The study by Lydell, Grahn, Baigi, Marklund and Mansson^16^ is in line with the findings of this study on age and gender as biographical predictors on a return-to-work decision with an addition of vocation (type of job). The person’s profession is another important factor for the ability to-return-to-work.

The employee may fear to-return-to-work due to the stigma related to depression, lack of work knowledge and skill and possibilities of secondary gain. The employer might also make it difficult to allow the occupational therapist to return the employee to work by ‘blocking’, ignorance or unwillingness to reasonably accommodate the employee, or by planning to dismiss the employee due to their previous poor work performance.

If the employee is unable to-return-to-work, the occupational therapist will need to determine the extent of the incapacity and whether the incapacity is temporary or permanent. The occupational therapist must not ignore the employee’s work environmental factors such as possibilities of retrenchment, dismissal and disciplinary hearing processes in facilitating the return-to-work process. There are no clear principles impacting the decision of returning the employee to work as it depends on a variety of factors.^7^

Declaring employees as permanently incapacitated is determined by the employee’s unresponsiveness to team treatment, which includes medication, individual and group therapy, persisting depressive symptoms for more than two years despite intervention and a high number of hospitalisation sessions without response. Permanent incapacity has a negative effect on an employee’s identity as a worker.^7^ Ross^7^ remarks that without a worker identity a person is unlikely to aspire to achieving a worker role, or they may clearly not be capable of returning to their former work role.

The employee’s level of motivation and action will ultimately impact on their ability to-return-to-work. Crouch and Alers^18^ observe that motivational problems impair a person’s will to even try to work. The employee’s motivation may be influenced by intrinsic or extrinsic motivators, level of creative ability and environmental factors at work.

## Conclusion

Work is considered to be valuable in a person’s life. MDD affects the employee’s ability to work. Given that occupational therapists can assess and intervene, they should be seen as crucial in the treatment of MDD as well as efforts to increase return-to-work rates. Occupational therapists ought to have sound knowledge, skill and experience in the area of work or vocational rehabilitation as well as mental health in order to formulate an appropriate return-to-work decision

It is, however, a challenging process for an occupational therapist to formulate a return-to-work decision because several factors, including the employee’s biographical profile, motivation, employer’s willingness and functional capacity evaluation results, will need to be taken into consideration.

Given the significant impact MDD has on the work performance of employees, the increasing numbers of adults living with MDD and the possible impact on lifestyle and, therefore, financial independence of those affected by MDD, it is important to contextualize the impact of the occupational therapist’s return-to-work decision within the MDGs. Researchers^4,5^ indicate in their work that mental health is not covered by the MDGs. However, there have been strong arguments put forward by Thornicroft^19^ as well as Votruba, Eaton, Prince and Thornicroft^20^ that mental health should be clearly included in the new Sustainable Development Goals which are currently being explored. The authors of this research would support this argument in view of South Africa’s socio-economic and political context. South African laws promote the continued employment of those living with injuries or illness, provided they meet the work requirements with, or without, work place adaptations or reasonable accommodations. Alignment with the MDGs would support the work of occupational therapists involved in the field of vocational rehabilitation.
